# Supplementary material for: Costs of cannabis testing compliance: Assessing mandatory testing in the California cannabis market
Source: PLoS One. 2020 Apr 23;15(4):e0232041. doi: 10.1371/journal.pone.0232041 (PMC7179872; doi:10.1371/journal.pone.0232041)
Supplement: S1 Appendix — (DOCX) [file pone.0232041.s001.docx]

**S1 Appendix**

The following set of questions aims to gather data to study the costs of cannabis testing compliance. In specific, we aim to collect financial, managerial, and logistics data from testing labs; as well as market prices for testing equipment, supplies and chemical reagents used by equipment, equipment running capacities, and any other testing inputs needed to build a compliant testing laboratory in California.

This interview is intended to be responded by licensed testing labs and related equipment suppliers.

**Confidentiality statement:** Our research group will use this information for exclusive research purposes. Neither private nor individual information provided by our interviewed will not be disclosed by any means.

**Set of questions:**

| 1. **Industry** |
| --- |
| 1. Please define a small, medium, and large size testing cannabis laboratory. According to your best guess, indicate the number of testing equipment, number of employees, testing capabilities, number of vehicles, square footage of buildings, etc. |
| - 1. Small size lab: |
| - 1. Medium size labs: |
| - 1. Large size labs |
| 1. Based on your knowledge of the industry, what is the current proportion of small, medium, and large-sized labs? |
| 1. **Sampling** |
| *We expect that laboratories will collect samples from distributors at different distances, depending on the number of distributors within an area.* |
| 1. What is the maximum distance between a laboratory and a distributor that you believe is sustainable to do business? 2. Do you think that the size of a lab would affect its capabilities to get samples from distributors located at longer distances? 3. Given the distance between labs and distributors, how many times per week does lab collect samples from a distributor? 4. Do you know if there are satellite labs that help to collect samples from distributors located farther away? If so, please describe how do they work. 5. What is number of distributors covered by that small, medium, and large size labs? 6. What is number of samplers in small, medium, and large size labs? 7. What is the most common batch size of cannabis and cannabis products? |
|  |
| 1. **Rejection rates** |
| 1. According with recent public reports, in the first two months of testing, nearly 11,000 samples have been tested and almost 2,000 failed (18%). Based on your knowledge of this current industry, what is your best guest of the current rejection rate? |
| 1. What is the most common test that fails passing samples? |
| 1. What is the most common cannabis or cannabis product that fails passing the test? |
| 1. What is the proportion of samples that are pre-tested? |
| 1. What is your best guess of the proportion of failed samples that remediate? |
| 1. What is your best guess of failure rates in remediated samples? |
| 1. Which type of test failures is more likely producer would attempt to remediate their batches? How? |
| 1. **Capital Cost**   *Note: Indicate all capital and other one-time costs associated with the implementation of your lab. For example, include specific testing equipment, start-up and support equipment, vehicles, ISO, IQ&OQ protocols, etc. If you do not feel comfortable disclosing the exact value, please provide us some reasonable range.* |
| **Select the type of your lab:**  Small-scale lab ________ Medium-scale lab________ Large scale-lab________   \| Name of equipment/ vehicle/ other \| Supplier/ brand/ version or model \| Number \| Type of analysis that can perform (if apply) \| Running time in minutes (if apply) \| Cost \| \| --- \| --- \| --- \| --- \| --- \| --- \| \|  \|  \|  \|  \|  \|  \| \|  \|  \|  \|  \|  \|  \| \|  \|  \|  \|  \|  \|  \| \|  \|  \|  \|  \|  \|  \| \|  \|  \|  \|  \|  \|  \| \|  \|  \|  \|  \|  \|  \| \|  \|  \|  \|  \|  \|  \| \|  \|  \|  \|  \|  \|  \| \|  \|  \|  \|  \|  \|  \| \|  \|  \|  \|  \|  \|  \| \|  \|  \|  \|  \|  \|  \| \|  \|  \|  \|  \|  \|  \| \|  \|  \|  \|  \|  \|  \| \|  \|  \|  \|  \|  \|  \| \|  \|  \|  \|  \|  \|  \| \|  \|  \|  \|  \|  \|  \| \|  \|  \|  \|  \|  \|  \| \|  \|  \|  \|  \|  \|  \| \|  \|  \|  \|  \|  \|  \| \|  \|  \|  \|  \|  \|  \| \|  \|  \|  \|  \|  \|  \| \|  \|  \|  \|  \|  \|  \| |
| 1. **Equipment costs of maintenance**   *Note: Indicate the annual cost of maintenance per testing equipment specified in section D. If you do not feel comfortable disclosing the exact value, please provide us some reasonable range.* |
| \| Name of the equipment \| Cost of annual maintenance \| \| --- \| --- \| \|  \|  \| \|  \|  \| \|  \|  \| \|  \|  \| \|  \|  \| \|  \|  \| \|  \|  \| \|  \|  \| |
| 1. **Annual costs of operations and maintenance**   *Note: Indicate all annual costs associated with operation and maintenance of your lab. For example, include the costs of compliance with ISO/IEC-17025 standards, the general maintenance, rent or mortgage, utilities, etc. If you do not feel comfortable disclosing the exact value, please provide us some reasonable range.* |
| \| Operations or maintenance \| Cost \| \| --- \| --- \| \|  \|  \| \|  \|  \| \|  \|  \| \|  \|  \| \|  \|  \| \|  \|  \| \|  \|  \| \|  \|  \| \|  \|  \| \|  \|  \| \|  \|  \| \|  \|  \| |
| 1. **Operational Cost of Equipment**   *Note: Indicate all consumable, such as reactive, gloves, glassware, etc. that are needed when running a specific equipment. If you do not feel comfortable disclosing the exact value, please provide us some reasonable range.* |
| \| Name of equipment \| Consumable associated \| Number or amount spend per minute \| Cost per unit \| \| --- \| --- \| --- \| --- \| \|  \|  \|  \|  \| \|  \|  \|  \|  \| \|  \|  \|  \|  \| \|  \|  \|  \|  \| \|  \|  \|  \|  \| \|  \|  \|  \|  \| \|  \|  \|  \|  \| \|  \|  \|  \|  \| \|  \|  \|  \|  \| \|  \|  \|  \|  \| \|  \|  \|  \|  \| \|  \|  \|  \|  \| \|  \|  \|  \|  \| \|  \|  \|  \|  \| \|  \|  \|  \|  \| \|  \|  \|  \|  \| \|  \|  \|  \|  \| \|  \|  \|  \|  \| \|  \|  \|  \|  \| \|  \|  \|  \|  \| \|  \|  \|  \|  \| \|  \|  \|  \|  \| \|  \|  \|  \|  \| |
| 1. **Labor Cost**   *Note: Per each position (e.g., manager, analyst, sampler, etc.), include salary and benefits, the number and the expected processing capacity. If you do not feel comfortable disclosing the exact value, please provide us some reasonable range.* |
| \| Position \| Number of employees \| Describe main duties \| Minimum requirements to get this position. Please, include years of experience if apply \| Processing capacity (samples per hour) \| Salary and benefits \| \| --- \| --- \| --- \| --- \| --- \| --- \| \|  \|  \|  \|  \|  \|  \| \|  \|  \|  \|  \|  \|  \| \|  \|  \|  \|  \|  \|  \| \|  \|  \|  \|  \|  \|  \| \|  \|  \|  \|  \|  \|  \| \|  \|  \|  \|  \|  \|  \| \|  \|  \|  \|  \|  \|  \| \|  \|  \|  \|  \|  \|  \| \|  \|  \|  \|  \|  \|  \| \|  \|  \|  \|  \|  \|  \| \|  \|  \|  \|  \|  \|  \| \|  \|  \|  \|  \|  \|  \| |
| 1. **Lab processing capacities** |
| **Please respond the following questions**   \| 1. Indicate the number of days per year that your lab operates   *If you can, include estimates for the other size categories (i.e., if your lab is a small size, include estimates for medium and large size labs)* \|  \| \| --- \| --- \| \| 1. Indicate the number of hours per day that your lab operates   *If you can, include estimates for the other size categories (i.e., if your lab is a small size, include estimates for medium and large size labs)* \|  \| \| 1. Indicate the percentage of downtime within a year   *If you can, include estimates for the other size categories (i.e., if your lab is a small size, include estimates for medium and large size labs)* \|  \| \| 1. Indicate the percentage of the processing testing efficiency   *If you can, include estimates for the other size categories (i.e., if your lab is a small size, include estimates for medium and large size labs)* \|  \| \| 1. Indicate the percentage of the running capacity   *If you can, include estimates for the other size categories (i.e., if your lab is a small size, include estimates for medium and large size labs)* \|  \| \| 1. Indicate the number of samples per day with a COA   *If you can, include estimates for the other size categories (i.e., if your lab is a small size, include estimates for medium and large size labs)* \|  \| |
