# Supplementary material for: Costs of cannabis testing compliance: Assessing mandatory testing in the California cannabis market
Source: PLoS One. 2020 Apr 23;15(4):e0232041. doi: 10.1371/journal.pone.0232041 (PMC7179872; doi:10.1371/journal.pone.0232041)
Supplement: Attachment — Submitted filename: Response to Reviewers.docx [file pone.0232041.s002.docx]

Dear Editor,

Thank you for offering us the opportunity to revise our manuscript for PLOS ONE. We have carefully revised the manuscript to address the comments of you and the reviewer and meet editorial guidelines.

Please see our responses in ***bold and Italic***.

Additional Editor Comments (if provided):

1. Please ensure that your manuscript meets PLOS ONE's style requirements, including those for file naming.

***We have revisited the PLOS ONE style templates and made the following changes:***

1. ***We have replaced the abbreviation of the state of California in authors’ affiliation.***

***We have included an indent at the start of a new section.***

2. ***We now cite figures as Fig***

2. We note that you have stated that you collected financial, managerial, and logistics data from managers or representatives of currently operating or prospective licensed testing labs. Please ensure that you have outlined the steps taken to ensure the data collected from these labs were suitably representative. Please include details of how the labs were selected and how many refused to provide their data or did not respond. Please also see our policy for citation of personal communications here: <https://journals.plos.org/plosone/s/submission-guidelines#loc-references>.

***We now have outlined the steps taken to collect data from representative labs and included the set of questions to which they responded. We have included a supplementary document (S1 Appendix) with the set of questions used as a guideline for the interviews. Within S1 Appendix, we describe the confidentially agreements discussed with interviewees in such a way that their names and details business information would not be disclosed. Please see Lines 230-241 of the document Revised Manuscript with Track Changes.***

3. We note that Figure 2 in your submission contains map images which may be copyrighted. All PLOS content is published under the Creative Commons Attribution License (CC BY 4.0), which means that the manuscript, images, and Supporting Information files will be freely available online, and any third party is permitted to access, download, copy, distribute, and use these materials in any way, even commercially, with proper attribution. For these reasons, we cannot publish previously copyrighted maps or satellite images created using proprietary data, such as Google software (Google Maps, Street View, and Earth). For more information, see our copyright guidelines: <http://journals.plos.org/plosone/s/licenses-and-copyright>.

We require you to either (1) present written permission from the copyright holder to publish these figures specifically under the CC BY 4.0 license, or (2) remove the figures from your submission:

1. You may seek permission from the original copyright holder of Figure 2 to publish the content specifically under the CC BY 4.0 license.

We recommend that you contact the original copyright holder with the Content Permission Form (<http://journals.plos.org/plosone/s/file?id=7c09/content-permission-form.pdf>) and the following text:

“I request permission for the open-access journal PLOS ONE to publish XXX under the Creative Commons Attribution License (CCAL) CC BY 4.0 (<http://creativecommons.org/licenses/by/4.0/>). Please be aware that this license allows unrestricted use and distribution, even commercially, by third parties. Please reply and provide explicit written permission to publish XXX under a CC BY license and complete the attached form.”

Please upload the completed Content Permission Form or other proof of granted permissions as an "Other" file with your submission.

In the figure caption of the copyrighted figure, please include the following text: “Reprinted from [ref] under a CC BY license, with permission from [name of publisher], original copyright [original copyright year].”

2. If you are unable to obtain permission from the original copyright holder to publish these figures under the CC BY 4.0 license or if the copyright holder’s requirements are incompatible with the CC BY 4.0 license, please either i) remove the figure or ii) supply a replacement figure that complies with the CC BY 4.0 license. Please check copyright information on all replacement figures and update the figure caption with source information. If applicable, please specify in the figure caption text when a figure is similar but not identical to the original image and is therefore for illustrative purposes only.

The following resources for replacing copyrighted map figures may be helpful:

USGS National Map Viewer (public domain): <http://viewer.nationalmap.gov/viewer/>

The Gateway to Astronaut Photography of Earth (public domain): <http://eol.jsc.nasa.gov/sseop/clickmap/>

Maps at the CIA (public domain): <https://www.cia.gov/library/publications/the-world-factbook/index.html> and <https://www.cia.gov/library/publications/cia-maps-publications/index.html>

NASA Earth Observatory (public domain): [http://earthobservatory.nasa.gov/](http://earthobservatory.nasa.gov/%20)

Landsat: <http://landsat.visibleearth.nasa.gov/>

USGS EROS (Earth Resources Observatory and Science (EROS) Center) (public domain): [http://eros.usgs.gov/#](http://eros.usgs.gov/)

Natural Earth (public domain): <http://www.naturalearthdata.com/>

***Figure 2 contains a map that we created using R software. R is free software, so as the packages that are needed to create figures and maps (e.g., ggplot, maps, etc.). We included a sentence in Lines 358-359 that explicitly indicates that the map has been created using R software and the zip codes published by the BCC. We have included the cites of these sources [15 and 16].***

4. Please ensure that you refer to Figure 5 in your text as, if accepted, production will need this reference to link the reader to the figure.

***We have included the reference and title of Figure 5. Please see Line 553 and Lines 564-565 with our edits.***

Reviewers' comments:

Reviewer's Responses to Questions

Comments to the Author

1. Is the manuscript technically sound, and do the data support the conclusions?

The manuscript must describe a technically sound piece of scientific research with data that supports the conclusions. Experiments must have been conducted rigorously, with appropriate controls, replication, and sample sizes. The conclusions must be drawn appropriately based on the data presented. 

Reviewer #1: Yes

2. Has the statistical analysis been performed appropriately and rigorously? 

Reviewer #1: Yes

3. Have the authors made all data underlying the findings in their manuscript fully available?

The [PLOS Data policy](http://www.plosone.org/static/policies.action#sharing) requires authors to make all data underlying the findings described in their manuscript fully available without restriction, with rare exception (please refer to the Data Availability Statement in the manuscript PDF file). The data should be provided as part of the manuscript or its supporting information, or deposited to a public repository. For example, in addition to summary statistics, the data points behind means, medians and variance measures should be available. If there are restrictions on publicly sharing data—e.g. participant privacy or use of data from a third party—those must be specified.

Reviewer #1: Yes

4. Is the manuscript presented in an intelligible fashion and written in standard English?

PLOS ONE does not copyedit accepted manuscripts, so the language in submitted articles must be clear, correct, and unambiguous. Any typographical or grammatical errors should be corrected at revision, so please note any specific errors here.

Reviewer #1: Yes

5. Review Comments to the Author

Please use the space provided to explain your answers to the questions above. You may also include additional comments for the author, including concerns about dual publication, research ethics, or publication ethics. (Please upload your review as an attachment if it exceeds 20,000 characters)

Reviewer #1: This is a wonderfully detailed study.

The idea to model sampling costs as a function of failure rates is direct and intuitive. Of course, tightening rules by lowering tolerance thresholds or adding prohibited substances will generally raise failure rates, and loosening rules would lower them.

The authors 1) model sample testing costs as a function of failure rates; 2) accounting for economies of scales, model costs for small, medium, and large laboratories; 3) assuming market shares of 70%, 20%, and 10%, for large, medium, and small laboratories, simulate average costs per sample, among many other model outputs.

At each step, models are very detailed, with generous information provided by the authors’ survey. For instance, laboratory economies of scale are generated considering variable failure rates, operational efficiency, and operating days per year. Ultimately large labs are modeled to have four times the cost of small labs but ten times the capacity, suggesting potential for the industry to consolidate.

This provides a general framework for the evaluation of cannabis testing costs, and I hope the authors’ model may be used as a basis for further research modeling the price effects of cannabis testing regulations.

It is interesting to wonder how this measure fares across states. What exactly would you be measuring: the competency of the laboratories, the stringency of the regulations, or the quality of the local product?

***We agree that this is an interesting question and should be subject to further research. In this article however, we focused on detailed analysis of data from California. We describe the cannabis testing regulations in other states that allow recreational cannabis in the “Background” subsection. We have added a sentence about cross-state comparisons in the brief discussion of further research in the concluding section.***

Minor notes:
“Costs needed to start a testing lab that meets California regulations depend [ON] the scale of the lab “ (p31). Insert “ON”.

***Thank you. We have included [ON] in Line 601of the document Revised Manuscript with Track Changes***

The authors compare testing standards for cannabis and food products. However, they do not seem to approach the question: is this an equal comparison? Does smoking or vaporizing a substance put the user at greater exposure to health risks than eating it? If somehow heating or combustion or exposure to the mouth, throat, and lungs puts a user at greater health risk, that may justify the more severe standards applied to cannabis.

***There may be higher risk of health impacts if a crop is contaminated with microbial or fungus and consumed via inhalers, we do not know if there is a different risk by consuming a product contaminated with pesticides. Cannabis and cannabis products are offered to be consumed via edibles or inhalable. Here we treat cannabis like any other crop in California, thus we do such a comparison. We have included the sentence in Lines 427-429 to make this explicit.***

The authors may consider also comparing adding tobacco standards to their comparison of cannabis and food products (or remarking briefly on the comparison), to provide a comparison of regulations pertaining to another combustible intended for inhalation.

***We did not elaborate on tobacco standards because tobacco is not a relevant crop for California (California accounts for an extremely low share of U.S. tobacco production. Of course, tobacco continues to be consumed and we have included a brief comparison between tobacco standards and cannabis (lines 627-631).***

6. PLOS authors have the option to publish the peer review history of their article ([what does this mean?](https://journals.plos.org/plosone/s/editorial-and-peer-review-process#loc-peer-review-history)). If published, this will include your full peer review and any attached files.


If you choose “no”, your identity will remain anonymous but your review may still be made public.


Do you want your identity to be public for this peer review? For information about this choice, including consent withdrawal, please see our [Privacy Policy](https://www.plos.org/privacy-policy).

Reviewer #1: Yes: Steven Davenport


[NOTE: If reviewer comments were submitted as an attachment file, they will be attached to this email and accessible via the submission site. Please log into your account, locate the manuscript record, and check for the action link "View Attachments". If this link does not appear, there are no attachment files to be viewed.]


While revising your submission, please upload your figure files to the Preflight Analysis and Conversion Engine (PACE) digital diagnostic tool, <https://pacev2.apexcovantage.com/>. PACE helps ensure that figures meet PLOS requirements. To use PACE, you must first register as a user. Registration is free. Then, login and navigate to the UPLOAD tab, where you will find detailed instructions on how to use the tool. If you encounter any issues or have any questions when using PACE, please email us at [figures@plos.org](mailto:figures@plos.org). Please note that Supporting Information files do not need this step.
